# Sex-Specific Prognostic Implications in Dilated Cardiomyopathy After Left Ventricular Reverse Remodeling

**DOI:** 10.3390/jcm9082426

**Published:** 2020-07-29

**Authors:** Antonio Cannata, Paolo Manca, Vincenzo Nuzzi, Caterina Gregorio, Jessica Artico, Piero Gentile, Carola Pio Loco, Federica Ramani, Giulia Barbati, Marco Merlo, Gianfranco Sinagra

**Affiliations:** 1 Cardiovascular Department, Azienda Sanitaria Universitaria Integrata di Trieste (ASUITS), University of Trieste, 34100 Trieste, Italy; anto.cannata@gmail.com (A.C.); paolo.manca91@yahoo.it (P.M.); vincenzo_nuzzi@libero.it (V.N.); Jessica.artico@hotmail.it (J.A.); pierogentile.87@gmail.com (P.G.); carola.pioloco@gmail.com (C.P.L.); fedi84it@yahoo.it (F.R.); gianfranco.sinagra@asuits.sanita.fvg.it (G.S.); 2 Department of Cardiovascular Sciences, Faculty of Life Sciences & Medicine, King’s College London, London SE5 9NU, UK; 3 Biostatistics Unit, University of Trieste, 34100 Trieste, Italy; caterina.gregorio@outlook.com (C.G.); gbarbati@units.it (G.B.)

**Keywords:** sex differences, dilated cardiomyopathy, left ventricular reverse remodelling, long-term outcomes

## Abstract

Background. Women affected by Dilated Cardiomyopathy (DCM) experience better outcomes compared to men. Whether a more pronounced Left Ventricular Reverse Remodelling (LVRR) might explain this is still unknown. Aim. We investigated the relationship between LVRR and sex and its long-term outcomes. Methods. A cohort of 605 DCM patients with available follow-up data was consecutively enrolled. LVRR was defined, at 24-month follow-up evaluation, as an increase in left ventricular ejection fraction (LVEF) ≥ 10% or a LVEF > 50% and a decrease ≥ 10% in indexed left ventricular end-diastolic diameter (LVEDDi) or an LVEDDi ≤ 33 mm/m^2^. Outcome measures were a composite of all-cause mortality/heart transplantation (HTx) or ventricular assist device (VAD) and a composite of Sudden Cardiac Death (SCD) or Major Ventricular Arrhythmias (MVA). Results. 181 patients (30%) experienced LVRR. The cumulative incidence of LVRR at 24-months evaluation was comparable between sexes (33% vs. 29%; *p* = 0.26). During a median follow-up of 149 months, women experiencing LVRR had the lowest rate of main outcome measure (global *p* = 0.03) with a 71% relative risk reduction compared to men with LVRR, without significant difference between women without LVRR and males. A trend towards the same results was found regarding SCD/MVA (global *p* = 0.06). Applying a multi-state model, male sex emerged as an independent adverse prognostic factor even after LVRR completion. Conclusions. Although the rate of LVRR was comparable between sexes, females experiencing LVRR showed the best outcomes in the long term follow up compared to males and females without LVRR. Further studies are advocated to explain this difference in outcomes between sexes.

## 1. Introduction

Dilated cardiomyopathy (DCM) is a heterogeneous primary muscle disease predominantly affecting men, with a male to female ratio 3:1. The prognosis of DCM has dramatically improved over the last decades [1,2,3] and the occurrence of left ventricular reverse remodelling (LVRR) under optimal medical treatment has been shown as one of the main prognostic drivers [1,4,5].

Female sex has recently emerged as an important outcome modifier in DCM patients, being independently associated with more favourable long-term outcomes and with a lower incidence of cardiovascular events in comparison to the male counterpart [6,7,8]. However, little is known regarding the mechanism underlying this important sex-specific effect. So far, none of the available reports have evaluated whether this difference could be partially explained by a different response to treatment and a more frequent occurrence of LVRR in women.

The aim of the present study was to investigate the rates of LVRR in males and females, and the prognostic impact of the relationship between LVRR and sex in a well-selected large cohort of real-world DCM patients with a long-term follow-up.

## 2. Methods

### 2.1. Study Population

All DCM patients consecutively enrolled in the Heart Muscle Disease Registry of Trieste between 1 January 1990 and 31 December 2015 and, with available data at 24-month follow up, were retrospectively analysed.

DCM was defined as an impairment of the Left Ventricular Ejection Fraction (LVEF) to < 50% and a left ventricular dilation in the absence of: a history of significant hypertension, obstruction > 50% of a major coronary artery branch, excessive alcohol intake, chemotherapy, an advanced systemic disease affecting short-term prognosis, pericardial diseases, congenital heart diseases, pulmonal, persistent supraventricular tachyarrhythmias, and active myocarditis [1,6].

The presence of a significant coronary artery obstruction was carefully excluded by a coronary artery angiography or, in case of a low likelihood of coronary artery disease, by coronary computed tomography scan.

All patients were on optimal medical treatment, receiving the highest tolerated doses of angiotensin-converting enzyme inhibitors or angiotensin receptor blockers and beta-blockers unless contraindicated [9]. Furthermore, implanted cardioverter defibrillators (ICDs) and cardiac resynchronization therapy (CRT) have been systematically introduced respectively since 1998 and 2005, according to international guidelines [10].

A structured outpatient follow-up, comprehensive of clinical evaluation, a 12-lead ECG, and two-dimensional echocardiography were performed at regularly scheduled time points until 24 months from enrolment (i.e., first evaluation at our Department) and then yearly or every other year afterwards according to specific clinical needs.

The institutional ethics board approved the study. The investigation complied with the Declaration of Helsinki.

### 2.2. Echocardiographic Evaluation

Left Ventricular (LV) dimensions and function were assessed according to international guidelines [11]. In particular, LV volumes and LVEF were calculated by Simpson’s biplane method, and all volumes were indexed according to body surface area. LV dilation based on LV end-diastolic volume was considered mild, moderate, or severe according to international guideline sex-specific reference values [11,12,13]. The LV filling pattern was classified as a restrictive filling pattern in the presence of E-wave deceleration time < 120 ms or E-wave/A-wave > 2 associated with E-wave deceleration time < 150 ms. Right ventricular dysfunction was defined as a right ventricle fractional area change (RVFAC) < 35%. Mitral regurgitation (MR) was considered significant only if moderate to severe.

### 2.3. LVRR Definition and Study Outcome Measures

LVRR was defined as an increase in the LVEF ≥ 10% (or LVEF > 50%) associated with a decrease ≥ 10% in indexed left ventricular end-diastolic diameter (LVEDDI) or (LVEDDI ≤ 33 mm/m^2^) at 24-month follow-up after enrolment, as previously described [5].

The main outcome measure was considered a composite of all-cause mortality, heart transplantation (HTx), and ventricular assist device (VAD) as destination therapy. A composite of sudden cardiac death (SCD) or major ventricular arrhythmias (MVA) was considered as the secondary outcome measure.

Specifically, MVA was defined as sustained ventricular tachycardia, ventricular fibrillation/flutter, or appropriate intervention of an ICD. SCD was defined as a death occurred within 1 h from the symptom’s onset, or as a death occurred during sleep in clinically stable patients with New York Heart Association (NYHA) class I–III.

To evaluate the association with the study outcome measures, the population was stratified into four groups, based on sex and the occurrence of LVRR.

Outcomes were investigated directly from the patient during the follow-up visit, medical records from the referral hospital or by telephone interview with the patient, relatives, or the general practitioner.

### 2.4. Statistical Analysis

Variables were expressed as mean and standard deviation, median and interquartile range (IQR), or counts and percentage, as appropriate. Comparisons between groups were made by the analysis of variance (ANOVA) test on continuous variables using the Brown-Forsythe statistic when the assumption of equal variances did not hold, or the nonparametric Mann-Whitney test when necessary; the chi-square test or the Fisher’s exact test were calculated for discrete variables.

Survival curves for the composite outcome measure of all-cause mortality/HTx/VAD were estimated and compared between groups by means of the Log-rank test. Cumulative incidence curves for the composite outcome measure of SCD/MVA were estimated and compared taking into account competing risks of death from other causes, and the appropriate statistical test suitable for competing risks was performed [14]. To investigate the impact of sex and LVRR on the outcomes, cause-specific multivariable Cox models were estimated from a list of candidate prognostic variables obtained from the univariable analyses (i.e., those with a *p*-value ≤ 0.1). For this analysis, the follow-up started after 24 months from enrolment, when the LVRR is considered to be completed [5]. Moreover, to further evaluate the relationship between sex and LVRR, a Markov illness-death model with all-cause mortality/HTx/VAD as absorbing state and the risk of LVRR as an intermediate state was estimated. The model consists of three discrete health states (i.e., alive without LVRR; alive with LVRR; dead or HTx or VAD) and a transition probability matrix (P) is calculated between states (see Supplementary Figure S1 for schematic representation). Specifically, a multi-state model fitting a Cox-type regression for each transition was used to estimate transition-specific hazard ratio (HR) for Sex. In this case, the follow-up started at the time of enrolment and this model was adjusted for a list of candidate variables significantly different at the univariable analysis of the multi-state model. The IBM-SPSS (New York, NY, USA) statistical software version 19 was used for descriptive analyses; the software R (R Foundation for Statistical Computing, Vienna, Austria. https://www.r-project.org/) was used for the cumulative incidence curves estimation (library “cmprsk”), to test the proportional hazards assumption for the Cox model and for the multi-state model (packages “ggplot2”, “survival” and “mstate”) [15].

## 3. Results

**Figure 1:**
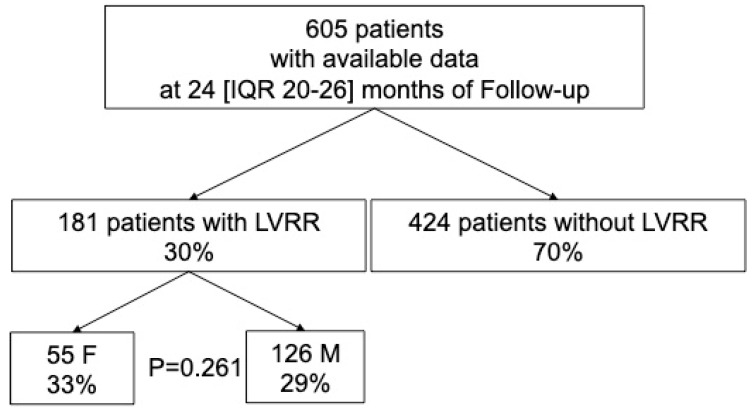
Diagram of study population. Legend. F: Females; LVRR: Left Ventricular Reverse Remodelling; M: Males.

A total cohort of 605 consecutive DCM patients with available data at a median follow-up of 24 (IQR 20–26) months was analysed (Figure 1). The main characteristics of the population at 24-month follow-up evaluation are summarized in Table 1. Patients were predominantly males (73% *n* = 440), and males were slightly younger than females (47 ± 15 vs. 51 ± 14 years respectively, *p* = 0.007). Females had a higher incidence of left bundle branch block (LBBB) compared to their male counterparts (34% vs. 25%, respectively, *p* = 0.02). All patients received optimal medical treatment, without differences between sexes.

### 3.1. Left Ventricular Reverse Remodeling

Overall, 30% of patients experienced a LVRR (*n* = 181), without significant differences between sexes: the cumulative incidence of LVRR at the 24 months evaluation was 33% in women vs. 29% in men (*p* = 0.26) (Figure 1). Indeed, the probability of undergoing LVRR was similar between men and women (Hazard Ratio for male sex [HR] 0.81, 95% Confidence Intervals [CI] 0.53–1.22, *p* = 0.31). Interestingly, at the 24-months evaluation, despite a comparable LVEF (40 ± 12% in women vs. 41 ± 11% in men, *p* = 0.32), women had a higher incidence of moderate to severe sex-specific LV dilation compared to men (59% vs. 28% respectively, *p* ≤ 0.001).

### 3.2. Outcomes

**Figure 2:**
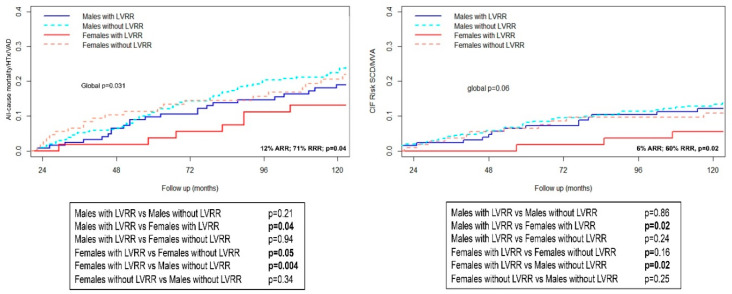
Kaplan-Meier curves for the incidence of All-cause mortality/HTx/VAD (Left Panel) and cumulative incidence function for SCD/MVA (Right Panel) according to LVRR and sex. Legend. HTx: Heart Transplantation; LVRR: Left Ventricular Reverse Remodelling; VAD: Ventricular Assist Device. MVA: Major Ventricular Arrhythmias; SCD Sudden Cardiac Death.

Overall, starting from the 24-months evaluation, the outcomes of women were more favourable compared to men (Figure 2). During a median follow-up of 149 (IQR 90–232) months, 189 patients (31%) experienced the main outcome measure (44 males with LVRR, 35%; 105 males without LVRR, 34%; 10 females with LVRR, 18%; and 30 females without LVRR, 27%; global log-rank *p* = 0.03) and 128 patients (21%) the secondary outcome measure (36 males with LVRR, 29%; 76 males without LVRR, 24%; 6 females with LVRR, 11%; 24 females without LVRR, 22%; global *p* = 0.06). The cumulative incidence at 10 years of follow-up of specific components of the outcome measure is reported in Table 2. Women experiencing LVRR had the lowest incidence of all-cause mortality/HTx/VAD at 10 years of follow-up compared to the other groups, with an absolute risk reduction of 12% and a relative risk reduction of 71% of the main outcome measure compared to men with LVRR (*p* = 0.04). Interestingly, women without LVRR at 24 months showed a similar incidence of adverse outcomes as males (Figure 2). Noteworthy, the cumulative incidence of arrhythmic events followed the same trend, being lower in women with LVRR than in the other subgroups (*p* = 0.06) with an absolute risk reduction of 6% and a relative risk reduction of 60% of the arrhythmic outcome measure at 10 years of follow-up compared to men with LVRR (*p* = 0.02) (Figure 2).

### 3.3. Multi-State Model Analysis

**Figure 3:**
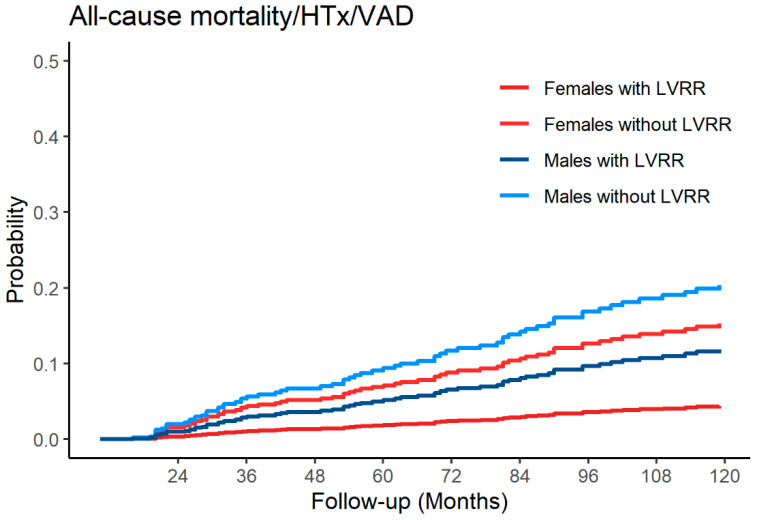
Adjusted Cumulative Incidence estimated from the multi-state model of All-cause mortality/HTx/VAD according to sex in patients with LVRR and without LVRR: Left Ventricular Re. Legend. HTx: Heart Transplantation; VAD: Ventricular Assist Device.

After adjustment for the different variables at the 24 months evaluation (i.e., Age, NYHA class, Sinus Rhythm, Severe LV Dilation, LVEF, Restrictive Filling Pattern, Right Ventricular Dysfunction, and medical therapy) male sex emerged as an independent risk factor of adverse outcomes (HR 1.86, 95% CI 1.07–3.82, *p* = 0.02). To further investigate the relationship between sex and the prognostic role of LVRR over time, a multistate model was built considering LVRR as an intermediate state, with the follow up starting from the baseline. The multi-state model highlights how the occurrence of LVRR over time was strongly associated with better outcomes (HR 0.01, 95% CI 0.001–0.04, *p* < 0.001) and male sex emerged as a strong prognostic factor in patients who experienced LVRR (HR 2.81, 95% CI 1.03–7.64, *p* = 0.04), whereas the impact of sex was diluted in patients without LVRR. Indeed, men with LVRR had a significantly higher probability of experiencing adverse outcomes over time (*p* = 0.04), whereas sex differences were blunted in those without LVRR over time (*p* = 0.52) (Figure 3).

## 4. Discussion

Female sex has emerged as an important outcome modifier in different cardiovascular scenarios. In patients with DCM, previous reports highlighted the protective role of female sex towards adverse outcomes over the long-term follow-up [6,7,8,16,17]. However, besides speculative hypotheses and observational analyses, there is no evidence so far investigating the possible mechanisms underlying this prognostic difference between sexes. Although one possible explanation might dwell in a different sex-specific response to medical treatment and, therefore, a different rate of LVRR with subsequent prognostic implications [5], evidence of that is still unavailable. To date, this is the first study addressing the interaction between sex and LVRR as potential outcome modifier in a large population of well-characterized DCM patients with available follow-up data.

The LVRR is a complex process that usually starts with the introduction of medical therapy and takes up to 24 months to complete [1]. Although several factors have been associated with the occurrence of LVRR over time [5], so far, little is known about the influence of sex on the rate of LVRR. Similarly to previous reports [5], in our population approximately 30% of patients experienced LVRR at 24 months of follow-up and the occurrence of LVRR was strongly associated with better prognosis (HR 0.01, 95% CI 0.001–0.04, *p* < 0.001). Interestingly and unexpectedly, the rate of LVRR was comparable between man and women (Figure 1). Noteworthy, among patients experiencing LVRR, females had an overall better prognosis compared to males during a very long-term follow-up; conversely a comparable prognosis between males and females without LVRR was found. To evaluate the prognostic impact of sex over time, we used a multi-state model considering the occurrence of LVRR as an intermediate state. The LVRR was confirmed as a long-term prognostic predictor and the female sex was strongly associated with better outcomes predominantly in patients experiencing LVRR whereas its prognostic implications were diluted in those not experiencing LVRR (Figure 3).

Despite the optimization of medical and device therapy, at 24-months revaluation women still showed a more advanced phenotype of the disease, characterized by larger LV diameters and a higher incidence of moderate to severe sex-specific dilation, which might partially justify the comparable outcomes in patients without LVRR (Table 1). Despite the more advanced phenotype of DCM observed at 24-month revaluation, women had overall better long-term outcomes than men. This was probably driven by the excellent long-term outcome showed by women experiencing LVRR, compared to either men with LVRR or patients without LVRR regardless of their sex (Figure 2). Indeed, women experiencing LVRR showed a 71% relative risk reduction of experiencing a composite adverse outcome of all-cause mortality/HTx/VAD compared to men with LVRR. Similar trends were found for arrhythmic events (Figure 2).

In the era of precision medicine, these findings might have important clinical implications, opening new possible scenarios in patients’ management. In fact, different treatment strategies might be employed between sexes experiencing LVRR or not over time. Our results highlight an independent prognostic role of female sex, especially after the LVRR is achieved, and opens up novel scenarios to investigate the mechanism underlying this prognostic advantage of women besides response to treatment.

### Women and DCM, a Fairy Tale?

**Figure 4:**
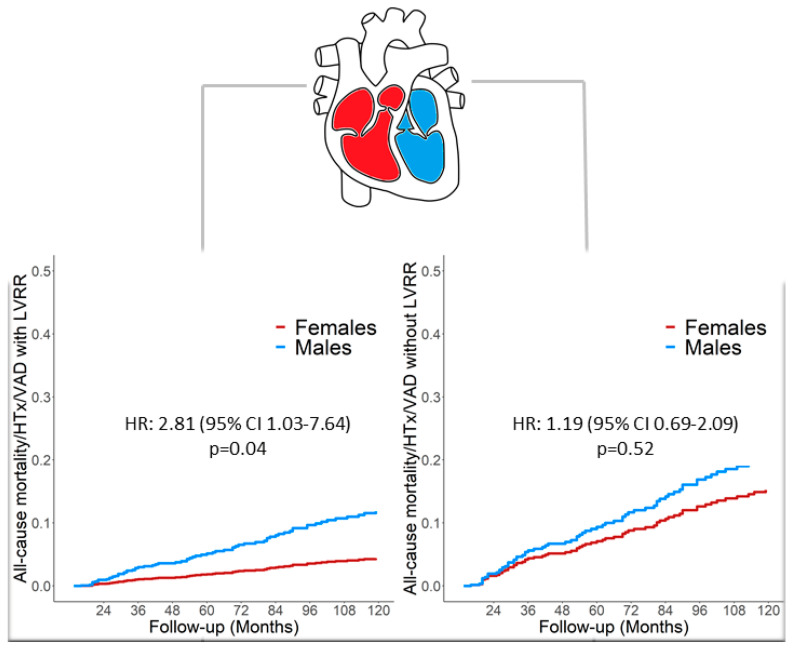
Central Illustration Schematic representation of the main results of the study. The rate of LVRR is comparable between sexes; however, male sex is an independent adverse prognostic factor regardless of the occurrence of LVRR. Legend: Legend. HTx: Heart Transplantation; LVRR: Left Ventricular Reverse Remodelling; VAD: Ventricular Assist Device.

The mechanisms behind a prognostic benefit of female sex are still largely unknown. Indeed, in large clinical trials, women showed a variable response to medical treatment whereas the benefit in men was clear-cut [18]. Furthermore, large registry analysis, probably due to the short-term observation provided, failed to demonstrate a prognostic advantage of female sex in heart failure (HF) patients [19]. Our results provide novel prognostic insights into sex differences in patients with DCM. In the present analysis, we demonstrated that, despite previous hypotheses, there is no difference in response to standard heart failure treatment between sexes, with a similar rate of LVRR over time. However, despite the comparable rate of LVRR, male sex was confirmed as an important independent adverse prognostic factor in those patients (Figure 4). This finding suggests that the reason for this prognostic benefit in women might dwell in some intrinsic factors specifically related to the female sex. Furthermore, potential and yet unknown protective mechanisms might be present in female patients with DCM helping either to control the occurrence or to suppress life-threatening arrhythmic events, highlighting the protective role of female sex also in this setting. Whether different social or cultural behaviours associated with hormonal status or genetic background might have a role in this is still largely unknown and deserves further study [18,20,21,22,23].

## 5. Limitations

This retrospective analysis has been conducted in patients with DCM consecutively enrolled in a tertiary referral centre. Therefore, these results might be not genuinely representative of the entire DCM spectrum and should be applied only to patients with similar characteristics. A possible selection bias imposed by the long enrolment period has been imposed by the relatively low event rate. However, guideline-directed medical treatment has been provided to all patients regardless of the date of enrolment, partially overcoming this limitation. Data on cardiac magnetic resonance, biomarkers and genetics were not available for all patients. Similarly, evaluation of the potential sex-specific effect of device therapy requires larger multicentre analysis. Therefore, limiting the investigation of these specific subgroups might introduce a significant bias in the population analysed. Lastly, sex-specific analyses on the occurrence of arrhythmic events are needed to provide more in-depth characterization of these patients. Further research is needed to confirm these data in larger multicentric populations, focusing on advanced imaging analysis and novel biomarkers or genetic status aiming to provide novel insights in this field.

## 6. Conclusions

In this large and well-selected cohort of patients affected by DCM, the rate of LVRR was similar between males and females. However, females achieving LVRR experienced a more favourable long-term prognosis and male sex has been confirmed as independently associated to adverse prognosis even after the LVRR is achieved. A precise characterization of DCM, including genetic background, will be essential to explain this difference in outcomes between men and women in the future.

## Figures and Tables

**Table 1:** Characteristics of the Population at 24-month evaluation.

	Total Cohort		
	605 Patients		
	Female	Male	*p*-Value
*n*	165	440	
Age, (mean ± SD)	51 ± 14	47 ± 15	0.007
SBP, (mean ± SD)	123 ± 18	127 ± 50	0.39
NYHA III/IV, *n* (%)	20 (13%)	34 (8%)	0.11
Familial History of DCM, *n* (%)	37 (23%)	109 (26%)	0.45
Sinus Rhythm, *n* (%)	138 (90%)	348 (86%)	0.19
LBBB, *n* (%)	52 (34%)	101 (25%)	0.02
QRS Length, (mean ± SD)	116 ± 35	115 ± 35	0.69
LVEDDI, mm/m^2^ (mean ± SD)	34 ± 5	31 ± 5	<0.001
LVEDVI, mL/m^2^ (mean ± SD)	78 ± 31	81 ± 32	0.42
Normal Volumes *, *n* (%)	50 (32%)	223 (53%)	0.001
Mild Dilation *, *n* (%)	13 (8%)	80 (19%)	
Moderate Dilation *, *n* (%)	37 (24%)	30 (7%)	
Severe Dilation *, *n* (%)	55 (35%)	89 (21%)	
Moderate-Severe Dilation, *n* (%)	92 (59%)	119 (28%)	<0.001
LVEF %, (mean ± SD)	40 ± 12	41 ± 11	0.50
RFP, *n* (%)	12 (11%)	28 (9%)	0.57
RV Dysfunction, *n* (%)	9 (8%)	39 (11%)	0.29
ACE-I/ARBs, *n* (%)	122 (82%)	343 (85%)	0.51
β-blockers, *n* (%)	135 (85%)	368 (87%)	0.41
MRAs, *n* (%)	22 (14%)	57 (14%)	0.89
ICD during follow-up, *n* (%)	39 (24%)	140 (32%)	0.06
CRT during follow-up, *n* (%)	19 (12%)	59 (13%)	0.59

* Gender specific volumes (LVEDV/BSA): Normal volumes Females: < 61 mL/m^2^. Males: < 74 mL/m^2^; Mild dilation Females: 62–70 mL/m^2^. Males: 75–89 mL/m^2^; Moderate Dilation Females: 71–80 mL/m^2^. Males: 90–100 mL/m^2^; Severe Dilation Females: > 80 mL/m^2^. Males: > 100 mL/m^2^. [10] Legend: ACE-I: Angiotensin Converting Enzyme-Inhibitors; ARBs: Angiotensin Receptor Blockers; BSA: Body Surface Area; CRT: Cardiac Resynchronization Therapy; ICD: Implantable Cardioverter Defibrillator; LBBB: Left Bundle Branch Block; LVEDDI: Left Ventricular End Diastolic Diameter Indexed; LVEDVI: Left Ventricular End Diastolic Volume Indexed; LVEF: Left Ventricular Ejection Fraction; MRA: Mineralocorticoid Receptor Antagonists; NYHA: New York Heart Association; RFP: Restrictive filling pattern; RV: Right ventricular; SBP: Systolic Blood Pressure.

**Table 2:** Cumulative incidence of events at 10 years of follow-up (starting from the 24 months evaluation) according to sex and LVRR.

	Male with LVRR	Male without LVRR	Females with LVRR	Females without LVRR
Median follow-up, months (IQR)	187 (122–269)	136 (80–202)	199 (123–278)	135 (72–222)
All-cause mortality/HTx/VAD	0.17	0.18	0.05	0.18
CV death/HTx/VAD	0.11	0.13	0.04	0.15
Death for pump failure	0	0.04	0.04	0.2
Heart Transplantation	0.05	0.05	0	0.10
VAD	0.007	0.003	0	0
SCD	0.06	0.03	0	0.04
SCD/MVA	0.10	0.12	0.04	0.12

Legend: CV: cardiovascular; HTx: Heart Transplantation; MVA: major ventricular arrhythmias; SCD: Sudden Cardiac Death; VAD: Ventricular assist device.

## Supplementary Materials

The following are available online at https://www.mdpi.com/2077-0383/9/8/2426/s1, Figure S1: The model consists of three discrete health states (i.e., alive without LVRR; alive with LVRR; dead or HTx or VAD) and a transition probability matrix (P) is calculated between states.
