# Supplementary material for: Sex-Specific Prognostic Implications in Dilated Cardiomyopathy After Left Ventricular Reverse Remodeling
Source: J Clin Med. 2020 Jul 29;9(8):2426. doi: 10.3390/jcm9082426 (PMC7464387; doi:10.3390/jcm9082426)
Supplement: Supplementary file 1 [file jcm-09-02426-s001.zip › jcm-870701-supplementary.docx]

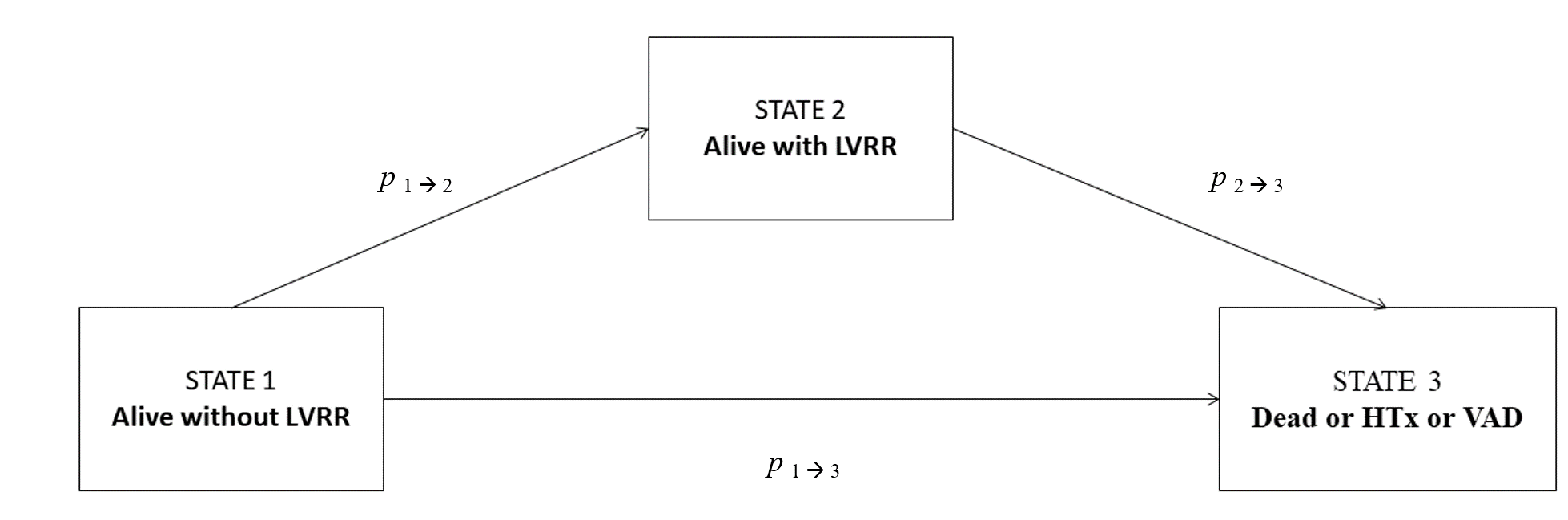


**Figure 1.** The model consists of three discrete health states (i.e., alive without LVRR; alive with LVRR; dead or HTx or VAD) and a transition probability matrix (P) is calculated between states.
